# Promoters of *Escherichia coli* versus *Promoter Islands*: Function and Structure Comparison

**DOI:** 10.1371/journal.pone.0062601

**Published:** 2013-05-22

**Authors:** Valeriy V. Panyukov, Olga N. Ozoline

**Affiliations:** 1 Department of Bioinformatics, Institute of Mathematical Problems of Biology, Russian Academy of Sciences, Pushchino, Moscow Region, Russian Federation; 2 Department of Functional Genomics and Cellular Stress, Institute of Cell Biophysics, Russian Academy of Sciences, Pushchino, Moscow Region, Russian Federation; 3 Department of Cell Biology, Pushchino State Institute of Natural Sciences, Pushchino, Moscow Region, Russian Federation; Center for Genomic Regulation, Spain

## Abstract

Expression of bacterial genes takes place under the control of RNA polymerase with exchangeable σ-subunits and multiple transcription factors. A typical promoter region contains one or several overlapping promoters. In the latter case promoters have the same or different σ-specificity and are often subjected to different regulatory stimuli. Genes, transcribed from multiple promoters, have on average higher expression levels. However, recently in the genome of *Escherichia coli* we found 78 regions with an extremely large number of potential transcription start points (*promoter islands*, PIs). It was shown that all PIs interact with RNA polymerase *in vivo* and are able to form transcriptionally competent open complexes both *in vitro* and *in vivo* but their transcriptional activity measured by oligonucleotide microarrays was very low, if any. Here we confirmed transcriptional defectiveness of PIs by analyzing the 5′-end specific RNA-seq data, but showed their ability to produce short oligos (9–14 bases). This combination of functional properties indicated a deliberate suppression of transcriptional activity within PIs. According to our data this suppression may be due to a specific conformation of the DNA double helix, which provides an ideal platform for interaction with both RNA polymerase and the histone-like nucleoid protein H-NS. The genomic DNA of *E.coli* contains therefore several dozen sites optimized by evolution for staying in a heterochromatin-like state. Since almost all *promoter islands* are associated with horizontally acquired genes, we offer them as specific components of bacterial evolution involved in acquisition of foreign genetic material by turning off the expression of toxic or useless aliens or by providing optimal promoter for beneficial genes. The putative molecular mechanism underlying the appearance of *promoter islands* within recipient genomes is discussed.

## Introduction

Bacterial transcription is carried out by a single enzyme DNA-dependent RNA polymerase (RNAP), which utilizes exchangeable σ-subunits to recognize and activate different promoter types. The genome of *E.coli* encodes seven σ-subunits [1], [2]. Alternative σ-factors are required to express a limited number of specific genes during normal growth (σ^FecI^, σ^F^ and σ^N^), and/or to survive in a variety of stress conditions (σ^S^, σ^H^ and σ^E^) [2]. Most bacterial genes are transcribed by the enzyme containing housekeeping σ-factor - σ^D^, which activates several thousand of promoters with certain correspondence to the consensus motifs TTGACA and TG-TATAAT, located about 35 and 15 bp upstream of the transcription start point (TSP), respectively. Sequence motifs recognized by RNAPs with alternative σ-factors differ from those of σ^D^ [3]–[9]. The difference is minimal for the σ-factor of general stress (σ^S^). That is why many σ^S^ promoters can be activated by the σ^D^-RNAP and *vice versa* [6]. In the case of σ^H^ and σ^E^ promoters (heat shock response) the difference is much more pronounced [7]–[9] but most of them can also be activated by σ^D^-RNAP [10]. This functional overlap, implying overlay of several promoters in one site, has been documented for at least one other pair of holoenzymes (σ^S^- and σ^H^-RNAP [11]). For all that, genes highly expressed in various growth conditions often contain several promoters with the same or different σ-specificity, which are regulated by different transcription factors (RegulonDB [12]). For example, the gene encoding σ^H^ (*rpoH*) is regulated by five transcription factors and can be transcribed from five closely spaced promoters, of which one combines σ^D^- and σ^S^-specificity, while others are activated by σ^N^-, σ^E^- and σ^D^–RNAP [8], [13]–[17]. Such multiplicity integrates genes into regulatory networks of bacterial cells and can be considered as a beneficial property. But only 24% of *E.coli* genes have two or more documented promoters, thus indicating a tendency to express genes from a single promoter.

The transcription start points of most known promoters have been mapped in *E.coli*, one by one, using classical biochemical approaches. Since the distance between the TSP and the −10 element in promoters varies from 2 to 11 bp, and RNA synthesis can be primed by 2–4 bases long primers [18], it is quite reasonable to expect certain distribution of registered TSPs around the optimal position. However, 2–7 start points in adjacent positions were observed only for 19% of promoters (analysis of our promoter compilations [19], [20]). Though high-throughput techniques gave higher percentage of promoters with multiple starts (30–39%) (analysis of the data [21], [22]), it is likely that genes prefer to contain only one functional promoter, which initiates transcription from a single TSP.

Clusters of potential promoters in bacterial genomes for the first time were discussed by Huerta and Collado-Vides [23]–[25]. Employing the position weight matrices generated by WCONSENSUS, the authors received on average 38 promoter-like signals within 250 bp upstream regions of genes if 3 standard deviations (StD) from the mean value of the promoter scores was allowed. To reduce the number of redundant signals, the authors introduced the “*external score*”, which took into account the position of the predicted TSPs relative to the initiating codon, and the “*cover function*” that allowed to ignore weak promoters in the vicinity of a stronger one [23]. However this improvement still left the number of predicted TSPs greater than the typical number of functional promoters (4.7 per region), and clusters of promoter-like sites were found for more than 80% of genes [23]. Since such clusters were not typical for coding sequences or intergenic spaces separating convergent genes, it was assumed that clusters of promoters predicted *in silico* should not be considered as merely false positives [23]–[25]. Additional promoters, for instance, can hold RNAP in transcriptionally inactive “closed” complexes, thereby increasing the enzyme concentration close to the real promoter. On the other hand, interaction of several RNAP molecules with overlapping promoter-like sites can interfere with normal initiation [26]. Perhaps the most intriguing is the assumption that additional promoter-like signals are “cryptic” promoters that are not active in a given genetic context, but can be activated by just one mutation, favoring adaptation of bacterial populations to environmental changes [25]. Here we discussed a possibility that extremely high density of potential promoters may be involved in the assimilation of foreign genes.

**Figure 1:**
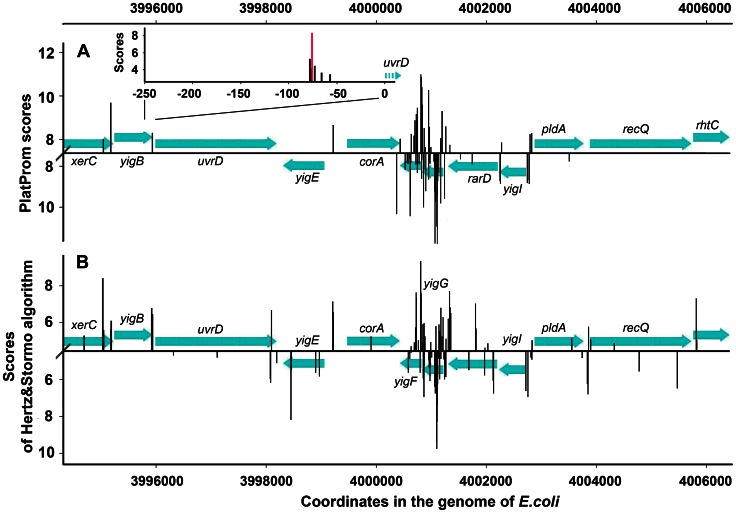
Distribution of the predicted promoters nearby *yigF-yigG* genes of *E.coli* MG1655. Positions of TSPs predicted by PlatProm (**A**) or by Hertz&Stormo algorithm [31] (**B**) are indicated by upward and downward vertical bars representing the values of scores for the top and bottom strands, respectively. The arrows show coding sequences of genes and directions of their transcription. X-axes in panels **A** and **B** correspond to 4 and 3 StD above the background level, respectively. The background level was defined as an average score of non-promoter DNAs (the set CS1 in [27]). The X-axis of the insert shows the distance to the initiating codon of *uvrD* and is placed at the level, which is 3 StD lower than the mean score within the set of *single* promoters (i.e. estimated as in [23] and [24]). The magenta bar corresponds to the experimentally mapped TSP [32].

Our promoter finder PlatProm [27]–[29] predicts on average 14 TSPs within 250 bp regulatory regions of genes transcribed from a single promoter, but on average 5.9 TSPs form an expected compact clusters near the position with the maximal score (exemplified in the insert of Figure 1A). That means that at a low cut-off level (3 StDs below the mean score of real promoters) we usually have 1 or 2 redundant promoter clusters per region. However, at a higher threshold (4 StDs above the background, or p<0.00004), PlatProm usually offers only one start point (Figure 1A), which in 83.1% of σ^D^-promoters either exactly fits to the experimentally mapped start or is located in a neighboring position (±2). However, some genomic loci not obey this rule and have extremely high density of potential TSPs. We named them *promoter islands* [27], [30], if PlatProm predicted at least 8 TSPs on any strand within every sliding window of 100 bp, and such abnormal density was observed for at least 300 bp (Figure 1A). In the genome of *E.coli* we found 78 such *islands* with length varying in the range 300–1101 bp and made sure that the high density of TSPs can not be an artifact of our software, since another promoter finder also revealed the same bunching (Figure 1B).

Previously it has been observed that the high frequency of PlatProm predicted TSPs may be used as a marker of long *genomic islands* (GI) containing alien DNA, and a new sliding window method GIST (Genomic-island Identification by Signals of Transcription) has been developed to find these regions [33]. In the chromosome of *E.coli* MG1655 GIST predicted 59 GIs, with the length of 4–15 kb and the average density of TSPs in the 4 kb sliding window at least 5 fold higher than in genome. Forty of them contained PIs, which have on average 7 fold higher promoter densities than *genomic islands*. It was proposed [33] that excessive promoters within *genomic islands* were emerged by accelerated evolution so as to integrate foreign genes into the host cells regulatory networks but according to the expression analysis performed on microarrays [34], most *promoter islands* are transcriptionally inactive [27], [30]. Thus; we compared *promoter islands* with normal promoters in terms of structural and functional properties in order to understand their biological role.

## Methods

### Analyzed sets of genomic samples

DNA fragments were taken from the chromosome of *Escherichia coli* K12 MG1655 (GenBank accession number U00096.2), where 78 PIs were identified by PlatProm [27]. Five additional sets, each of 78 samples, were collected for structural and functional comparison (Table S1).

- The *control* set was composed of genomic regions with minimal PlatProm scores, which were taken from the previously used compilation of non-promoter DNAs CS1 [27].
- The set of *single* (within 300 bp) promoters was collected using information of RegulonDB [12]. Promoters with the highest PlatProm scores were selected among other candidates.
- Samples with *multiple* promoters included at least 3 experimentally mapped TSPs within 300 bp.
- To compose the set of H-NS binding sites, we first used the chip-on-chip data published by Kahramanoglou et al. [35]. The authors provided 4 lists of genomic regions interacting with H-NS in different growth conditions. Sites overlapping with PIs were removed, and the set of 230 candidates, whose binding with H-NS was observed in all 4 experiments, was obtained. It was filtered using chip-on-chip data of Grainger et al. [36], [37] so as to collect 78 genomic regions with the highest H-NS binding capacity. Their nucleotide sequences were analyzed using the pattern matching tool *Virtual Footprint* (http://prodoric.tu-bs.de/vfp/vfp_promoter.php) [38] in order to find the H-NS binding modules, and 300 bp fragments with the center in the positions with the highest *Virtual Footprint* scores were chosen for structural analysis.
- Normal promoters of *alien* genes were selected from within the “*genomic islands*” identified in the *E.coli* DNA [33] by IslandViewer [39]. This software combines three different methods of finding long clusters of foreign genes relying on codon usage specificity and dinucleotide bias, but does not use transcription signals. Thirteen of the 32 “*genomic islands*” found by IslandViewer included PIs, so they were ignored. The remaining 19 “*islands*” contained only 18 experimentally mapped TSPs, but 3 of them have already been selected for other promoter sets. The missing 63 promoters were added using RegulonDB information on the *E.coli* transcription units. The centers of selected fragments were placed in PlatProm-predicted TSPs. Twelve of them were later mapped in RegulonDB with a 0–3 bp shift and in 7 cases the predicted starts deviate from the novel experimental TSPs for 8–16 bp. Functionality of 34 *alien* promoters was therefore confirmed experimentally.

### Chromatin immunoprecipitation data analysis

Association of RNAP and other DNA-binding proteins with *promoter islands* and normal promoters was assessed using chip-on-chip data [34]–[37], [40], [41]. The binding efficiency of RNAP was expressed as log_2_ of the ratio of hybridization signals obtained with DNA co-immunoprecipitated with the enzyme by σ^70^-specific antibodies and the control DNA recovered from the complexes without specific immunoprecipitation (experiment B in ref. 34). An ability of PIs and normal promoters to form complexes with nucleoid proteins H-NS, Fis, IHF and with the transcription factor FNR was investigated using data processed by authors. In the case of PIs the binding sites of different proteins were looking for within their boundaries (Table S1). For *single* promoters and promoters of “*genomic islands*” the ±150 bp area around the experimentally mapped or predicted TSPs was searched. In the case of *multiple* promoters binding sites of proteins were searched in the area located between the position lying 150 bp upstream of the first TSP and position located 150 bp downstream of the last TSP. In the set of data published by Grainger et al. [36], [37], the genomic regions were considered as targets for interaction with proteins if they contained at least one probe with hybridization signals ratio ≥1.5. Three other data sets [35], [40], [41] provide genomic coordinates of regions occupied by the specific protein, so PIs and promoter regions were considered as targets for interaction if they overlap with the published binding sites for at least 20 bp in at least one experimental series.

### Differential expression analysis

Transcriptional activity of PIs was addressed using 5′-end-specific RNA-seq data (supplement in ref. [22]). Registered sequence reads were ascribed to genomic positions by the specially designed software RNAMatcher that determined the number of similar samples, searched for genomic regions perfectly matching to the 5′-terminal oligonucleotide of a given size and provided a report of multiple correspondence, if any. First we collected samples fully identical to the sequences of genomic DNA (44 bases in length) and plotted their distribution in the chromosome of *E.coli*. If the sample had several matches in the genome (for instance, the products of seven ribosomal operons), we assumed that all they gave equal contribution to the registered number of sequence reads. This number, thus, was divided by the number of matching sites, and the resulting quotient was ascribed to each of them. The sequence reads remained after the first selection, were subjected to the next round of search for samples having 43 bases perfectly matching the genome at the 5′-end, and so on. The next nucleotide within a sample collected at each step can be either mismatch or the first nucleotide of the adapter sequence. In the former case the sample may represent a normal transcript, while in the later one – short oligonucleotide. Efficiency of productive transcription was assessed by the number of samples with perfect compliance with the genome (44 bases); while the number of short oligos was estimated using samples containing adapter sequence GATCGTGACTG.

### Structural analysis

**Figure 2:**
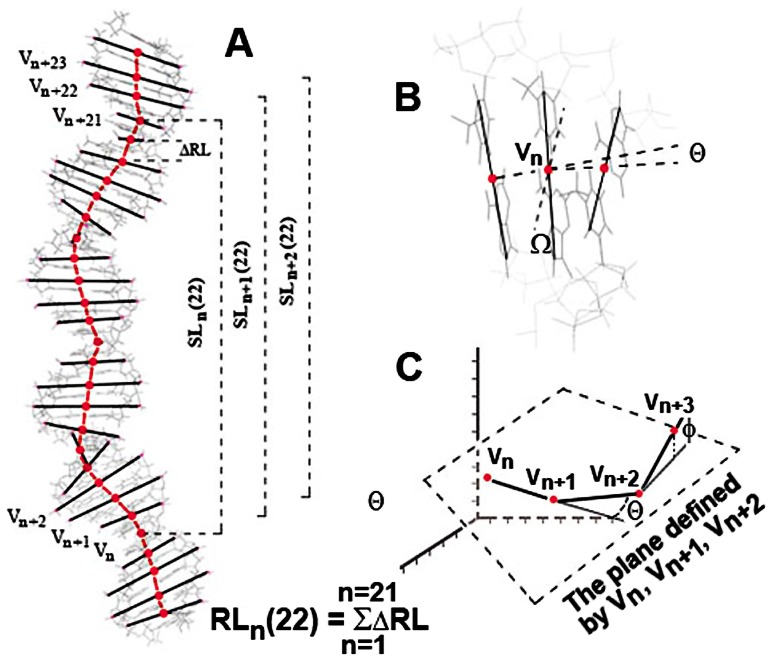
Schemes illustrating the metric parameters used. The 3D structure of 30 bp DNA fragment build by *DNA tools* [42] is exemplified in the panel A. The broken magenta line depicts the *phosphorus chain* with the dot vertices, where every one is the midpoint of the phosphorus doublet, shown as the black straight line segment. The chain length gives the parameter RL. The parameter SL measures the distance between the endpoints of the *chain*. The panel B illustrates the bending angle θ and the twist angle Ω at the vertex V_n_ of the *carbon chain*, where vertices show midpoints of the main base pair axes, connecting C_6_ of pyrimidines with C_8_ of purines. The panel C shows the torsion angle φ. While θ measures the bending of the chain in a plane passing through the vertices V_n_, V_n+1_, V_n+2_, the torsion angle φ reflects local unflatness of the chain.

3D models of DNA fragments (each 300 bp) were obtained in pdb-format by software *DNA tools* ([42], http://hydra.icgeb.trieste.it/dna/model_it.html) using electrophoretically estimated structural parameters of dinucleotides. These models were analyzed by the software aSHAPE [43], which used coordinates of the specific atomic groups to calculate the coordinates of the vertices of *conformational chains* (Figure 2). We employed *carbon* and *phosphorous* chains. The vertices of a *carbon* chain corresponded to the midpoint of a straight line connecting C_6_ of pyrimidines with C_8_ of purines (Figure 2B). In the B-form DNA the *carbon* chain reflects the curvature of DNA double helix. Configuration of the sugar-phosphate backbone was assessed by *phosphorous* chains, whose vertices corresponded to the midpoints of segments connecting phosphorus atoms of each complementary base pair (Figure 2A). The origin of a reference frame for each considered 3D fragment was imposed to the first vertex of employed chain.

Several structural parameters of chains were measured in order to assess the global and local conformation of DNA fragments. The global conformation was characterized by *Real Length* (**RL**), *Straightened Length* (**SL**) and cumulative twist angle (**Ω**), while the local conformation was defined by the bending angle **θ** and the torsion angle **φ** (Figures 2B and C).

**RL** was calculated as the total length of segments joining the vertices of *conformational chains*, while **SL** as the distance between two endpoints of the *chain* (Figure 2A). These two parameters reflected the global curvature of the given DNA fragment. **Ω** measured the angle between the long axes of two adjacent base pairs [44], or between two lines passing through their phosphorus atoms. The cumulative twist angle was calculated for fragments of a given size by simple summation.

The thermodynamic stability of DNA samples was characterized by stacking energy, which was calculated for fragments of different lengths by using the values computed for dinucleotides (DiProDB, http://diprodb.fli-leibniz.de/ShowTable.php) [45], [46].

## Results

**Figure 3:**
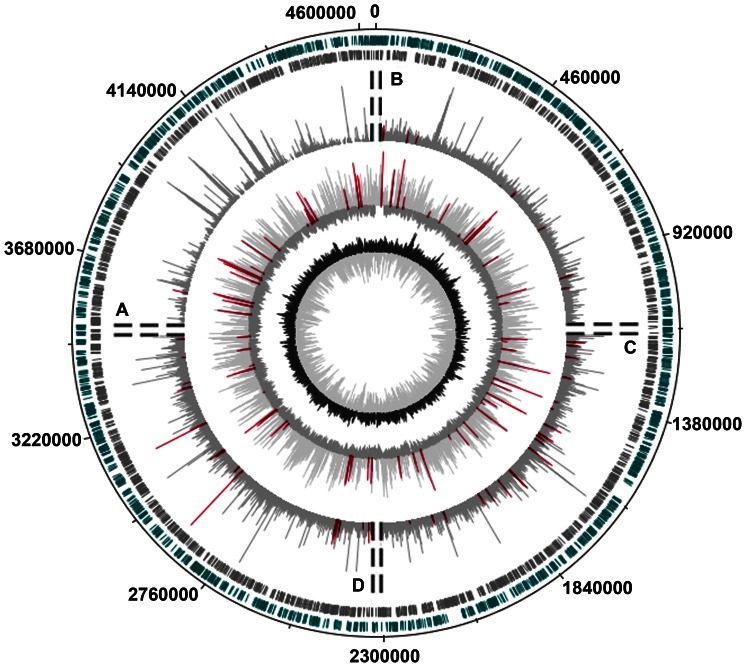
Distribution of transcriptional activity and RNAP binding sites in the genome of *E.coli* K12 MG1655. The genomic map was created with the DNAPlotter [47]. The **1-st** and the **2-nd** outer circles show the distribution of genes transcribed from different strands. The **3-rd** circle shows results of differential expression analysis (RNA-seq data [22]) reflecting the presence of different in size RNAs (plotted as log(N+1), where N is the number of registered sequence reads). The DNAPlotter sliding window was 10 bp, step – 10 bp. Since there was no asymmetry in the transcriptional activity of PIs along the genome, the results obtained for different in length RNAs were shown on one circle divided into 4 sections. **A**: 44 bases - long sequence reads (productive synthesis); **B**: samples, which sequences at the 5′-ends match the genome for at lest 14 or 13 bases; **C**: the same as **B** for 12 and 11 bases; **D**: the same as **B** for 10 and 9 bases. Sequence reads with multiple matching to the genomic DNA were taken into account. The **4th** circle shows the distribution of RNAP binding sites (log_2_ ratio of hybridization signals, window 300 bp, step 300 bp), revealed by the chip-on-chip technique (experiment B in ref. [34]). Magenta bars mark products transcribed from PIs and hybridization signals within PIs. The central circle shows the local GC-content (window size 300 bp, step 300 bp).

Our attention to *promoter islands* arose mainly because of the amazing combination of their functional properties. From the very beginning it was clear that PIs can interact with RNAP (circle 4 in Figure 3 and [27]) and undergo transition into the “open” state [27], [30], although the percentage of PIs that initiate RNAs detected by microarrays was significantly lower (∼13%) than in the case of normal promoters (59%) [27]. Based on the high density of promoter-like sites we hypothesized that proteins of transcription machinery constantly cover PIs and prevent the synthesis of normal mRNAs by hampering the “*promoter clearance*”. Short abortive RNAs must be synthesized in this case, but being bad templates for reverse transcription and subsequent amplification, they were undetectable on microchips. In this study we used RNA-seq data [22], which provided sequences of the 5′-ends of *E.coli* RNAs and contain samples, reflecting the number of even very short oligos in bacterial cells. To prepare the cDNA library authors extracted RNAs from rapidly growing cells, converted 5′-end triphosphates to monophosphates by tobacco acid pyrophosphotase and ligated them to 33 nucleotides long adapter. Modified RNAs were then reverse transcribed with a 26 nucleotides long primer, containing 9 random bases at the 3′-end. cDNAs flagged on both ends by known adapters, were amplified with primers without random sequences, and ∼80–200 bp long samples were gel purified. They contained 36 base pairs derived from primers and variable in length target sequences. Before sequencing samples were again amplified using longer primers (58 and 51 nucleotides). Ideally they mark both ends of initial transcripts, which minimal length expected to be 44n. As a result, 8,967,903 forty-four nucleotides long sequence reads were published by Dornenburg et al. [22] as raw data. Luckily, about half of them have the adapter sequence at the 3′-ends, which reflects the presence of short RNAs in the transcriptome. Their presence in the gel purified set may be explained by rather high diffusion capacity of short DNA fragments or by their base pairing with longer amplicons. In any case, it provided a unique opportunity to estimate quantity of short RNAs produced from different genomic regions.

### *Promoter islands* produce short oligonucleotides

Samples that have no adapter sequences and perfectly match to the genomic DNA, we considered as products of full-fledged transcription. The section **A** in Figure 3 shows relative amount of “long” RNAs, transcribed from the last quarter of *E.coli* genome. This part contains 25 out of 78 PIs, but only one of them, located in the promoter region of divergently transcribed genes *yhiL* and *yhiM* (positions 3,632,424–3,632,872), gave 10 products to the set. In the whole genome there are only 7 PIs that gave 88 sequence reads, if only internal regions of PIs were analyzed (Figure 3), or 10 PIs that gave 153 samples, if ±50 bp flanking regions were also taken into consideration (Figure S1). In the case of 78 *single* or 78 *multiple* promoters, this contribution was much higher (2,469 or 6,034 sequence reads, respectively), and detected samples were derived from 35 *single* or 51 *multiple* promoters. Thus, PIs are defective in productive transcription, supporting our previous observation made on the basis of microarray data [27].

Then we removed perfectly matching samples from the data set and looked for reads that have 43 bases long sequences at the 5′-end ideally matching to the genome and repeated this procedure for sequences of length 42, 41 … 9 bases. The relative amount of reads derived from PIs remained almost constant for samples corresponding to 44–15 nucleotides long RNAs and started to grow when this length decreased to 14 bases and less (Figure 3 sections B, C and D). Thus, it was likely that PIs can produce short oligos.

**Figure 4:**
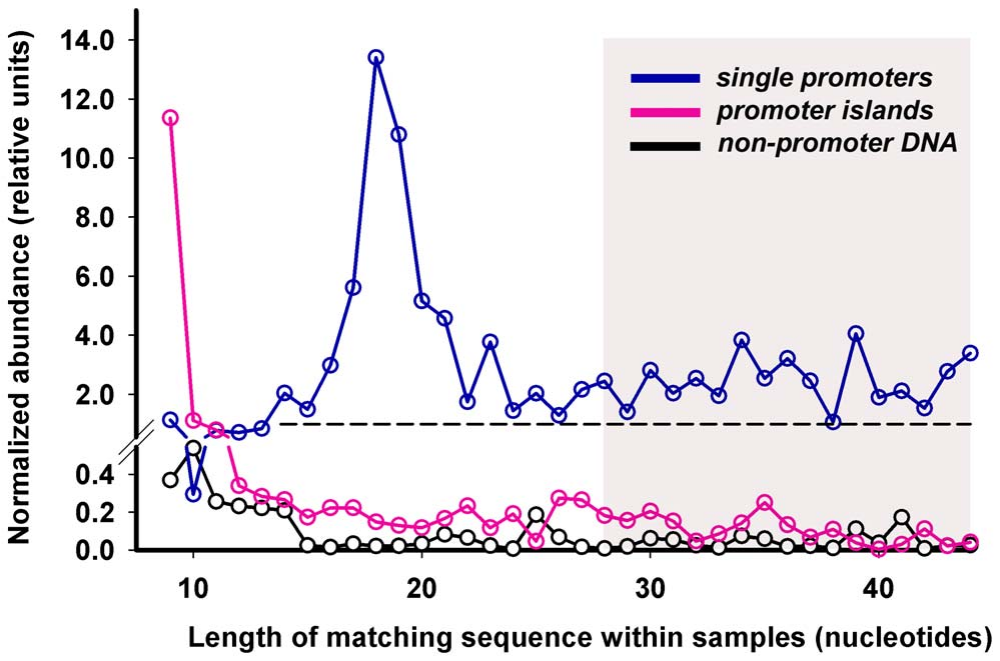
Relative amount of RNAs of different lengths in the cells of *E.coli* K12 MG1655. Samples containing at the 5′-end sequences matching genomic DNA for indicated length were collected step by step, as described in Methods and in the text. At the first 17 steps matching samples were collected from the whole set of registered sequence reads [22] (shaded area), while at the steps 18–36 – from samples containing the 3′-end adapter sequence. For *single* promoters samples were collected within ±50 bp regions surrounding TSPs; for each PI – within the area, covered by the *island*. For 78 non-promoter DNAs samples were collected within 300 bp long selected areas. The number of samples collected at each step for a particular set of genomic regions was normalized per the total number of sequence reads analyzed at this step, and per the total length of genomic regions in a set. In the case of random distribution it will give a value equal to 1.0 (dashed line). To increase the resolution in the bottom part of the figure we changed the scale of the Y-axis at the level 0.54.

However, the number of short products ascribed to *promoter islands* by this way may be overestimated, because some contribution to the set can gave samples derived from other genomic regions, which have sequences coinciding with PIs (the case of multiple matches). That is why; we reanalyzed the data using the samples with unique matching to the genome. Mismatches near the 5′-ends of long RNAs also increased the number of products erroneously considered as short RNAs. Though their contribution to the transcriptional output of *islands* should be much less than to the set of RNAs derived from normal promoters, we reduced this source of errors collecting 9–27 nucleotides long products from the subset of reads with adapter sequence at the 3′-end. As a result we observed the same dependence (Figures 4 and S1). Transcription output of PIs (magenta curves) was almost at the background level (black curves) and exceeded the average level (dashed line) only for samples with 9–10 matching bases. Activity of both *single* and *multiple* promoters was much higher (blue curves) showing no increase in the range of abortive RNAs.

In the case of *single* promoters (Figure 4), samples, corresponding to 17–19 nucleotides long oligos, gave a peak, probably reflecting the presence of microRNA-like RNAs that were recently found in *Streptococcus mutans* (typical size 16–26 bases) [48]. In the case of *multiple* promoters (Figure S1) we also observed a peak corresponding to longer RNAs (25–31 bases), which resemble “transcription start site associated RNAs” (tssRNAs) found in *Mycoplasma pneumoniae* [49]. Having an average size of 45 bases they were discussed as typical by-products of active promoters. PIs produce much less RNAs of this size and clearly differ in the pattern of transcription output from normal promoters.

Thus, PIs are transcriptionally competent *in vivo*, but the synthesis of long RNAs from these regions for some reason is quenched. We first assumed that this silencing is caused by interference between several RNAP molecules interacting with overlapping promoter-like sites. In this case, the 3D structure of PIs and normal promoters should be similar. Comparative analysis of their virtual models was undertaken to verify this assumption.

### 3D models of the *promoter islands* differ from that of normal promoters

At the first step 3D structures of PIs were compared with those of *multiple* promoters, which were assumed to be the better models of PIs than *single* promoters and with control non-promoter DNA fragments. Virtual 3D models were created by the “*DNA tools*” software [42], and the home package aSHAPE [43] was used for their analysis. Though the length of PIs varies in the range 300–1101 bp, equal in size fragments (300 bp) were selected so as to seize the part with the largest number of overlapping promoters.

Structural parameters used for comparison were chosen so that they reflected the properties associated with promoter function. Thus, it is generally assumed that the transcription complex formation is accompanied by DNA “wrapping” around the RNAP molecule [50], and properly located intrinsic bends in the DNA double helix facilitate this transition [51]. The difference **RL-SL**, which gave a measure of global curvature (Figure 2A), and two angles (**θ** and **φ**) representing local bends (Figures 2C and B), were therefore used to characterize the shape of DNA fragments. On the other hand, the initiation of RNA synthesis requires local DNA melting, which is hampered by supercoiling and a high stability of the double helix. Thus, we calculated the cumulative twist angle **Ω** nd stacking energy in order to estimate transition ability of modeled molecules.

**Figure 5:**
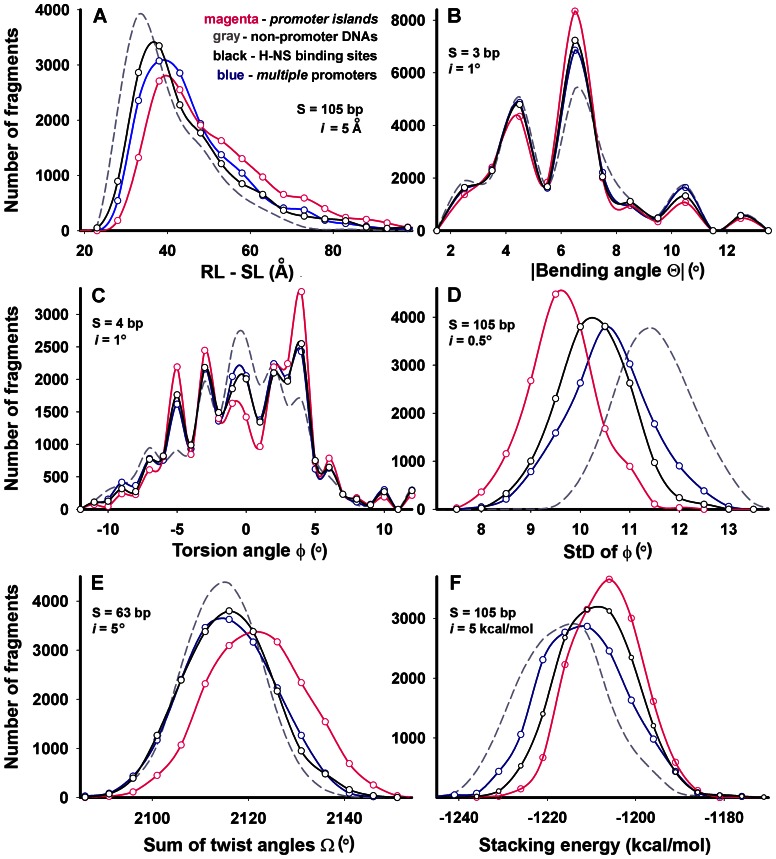
Histograms representing results of structural analysis. Metric parameters (specified under X-axes) were obtained using the *carbon* (**B**, **C** and **D**) or *phosphorus* (**A**, **E**, and **F**) *chains*. Studied genomic regions and the colors used are indicated in the panel **A**. The number of fragments that have similar values of the measured parameters were combined in the intervals “***i***”, which are indicated in panels. Parameters **RL-SL**, **Ω** and stacking energy were measured for fragments of different lengths in the range 20–200 bp. Observed dependences were exemplified for fragments of indicated length (**S**).

For comparison 300 bp 3D models were transformed into a set of short fragments of a given size (**S**) using the mode of sliding window. Figure 2A illustrates this procedure for 22 bp fragments, collected within the molecular model of 30 bp. In a molecule of 300 bp it gave 300−22 = 278 metric values, and 278×78 = 21,684 characteristic values for the entire set. Histograms exemplifying the data obtained for PIs, *multiple* promoters and control samples are shown in Figure 5.

We found that *multiple* promoters (blue curves) differ from non-promoter DNA (dashed gray curves) in all parameters tested except twist angles (Figure 5E), while PIs (magenta curves) differ from normal promoters in all metrics. The set of PIs, for instance, contained more fragments with large values of **RL-SL** (Figure 5A), which indicates their greater curvature. This bending can not be explained by the higher average value of **Θ**, because it was almost the same for all sets (5.5–5.6°) (Figure 5B). However the torsion angle **φ** most probably contributed to this difference, because the number of tetramers with almost zero **φ** in *promoter islands* was noticeably lower than in other two sets (Figure 5C). Moreover, the variations of **φ** were minimal for PIs (Figure 5D), which assumes certain structural regularity. PIs are on average more twisted (Figure 5E). Decreasing the negative supercoiling of natural DNA, this can complicate its local melting required for the transcription initiation. But the stacking energy (Figure 5F), measured for fragments of different sizes; as well as a higher AT-content of PIs (71.2% versus 58.3% for *multiple* promoters and 44.9% for non-promoter DNAs, the central circle in Figure 3) by contrast, showed less stability of DNA double helix, which promotes the formation of an open complex.

If the greater difference from control samples observed for PIs than for *multiple* promoters is simply due to the larger number of potential RNAP binding sites, curves reflecting structural features of *single* promoters should be shifted towards curves of control samples. This was really the case for **RL-SL**, the bending angle **Θ** and the torsion angle **φ**, while other metrics remained almost unchanged (Figure S2) excluding a possibility to explain the difference between normal promoters and PIs by the different number of RNAP binding sites. Small variations of **φ**, large values of **Ω** and values of stacking energy presumed evolutionary optimization of PIs for some other biological function(s), in addition to RNAP binding.

### H-NS is specifically involved in complex formation with *promoter islands*

**Figure 6:**
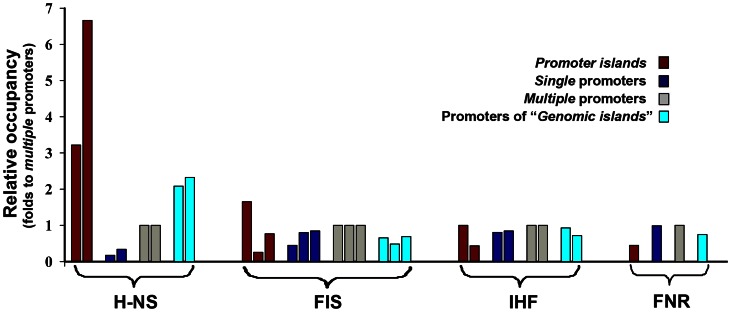
Relative occupancy of PIs and normal promoters by DNA-bound proteins. The plot shows the relative number of PIs (magenta bars), *single* (blue bars) and *multiple* (gray bars) promoters, as well as promoters of alien genes (cyan bars) involved in interaction with H-NS, Fis, IHF and FNR according to the published chip-on-chip data [34]–[36], [40], [41]. First we evaluated the percentages of PIs and promoters of all categories, which interacted with a given protein, and then expressed them as fold ratio to the percentages of *multiple* promoters. The first bar in each group represents the data obtained for cells grown in Luria-Bertani medium [35], [40]; the second bar – M9 medium+fructose [36], [37]; the third bar on the Fis plot - M9 medium+glucose [41]. FNR binding was assessed using the cells grown anaerobically in M9 medium+fructose up to the mid-log phase [37]. Publications [35], presented data obtained from cells harvested at different growth stages. We combined them in order to account all the binding sites.

Suppressed transcriptional activity of *promoter islands* suggested their existence in heterochromatin-like state, which is usually mediated by the specific proteins of bacterial nucleoid. Thus, we compared occupancy of PIs and normal promoters by nucleoid proteins H-NS, Fis and IHF using available chip-on-chip data [35]–[37], [40], [41]. In order to juxtapose the data obtained by different research groups, occupancies of *multiple* promoters were used for normalization (Figure 6).

Analyzed data sets testified almost equal interaction of Fis and IHF with *single* promoters (blue bars). Their interaction with PIs (perhaps due to differences in experimental conditions) was variable (magenta bars) but an average portions of PIs recruiting H-NS were comparable with *single* promoters. So we had no reason to consider these proteins as specific silencers of PIs. We did not notice essential difference in the ability of PIs to bind transcriptional regulators CRP, FNR, LexA or RutR (for FNR exemplified in Figure 6). However all data sets indicated a very high ability of PIs to form complexes with H-NS (Figure 6 and Table 1, columns **H**). According to the data published by Kahramanoglou et al. [35] the total area occupied by H-NS within *promoter islands* in different growth conditions varied in the range 24729–30026 bp (expected by chance 4661–6784 bp), i.e. up to 90% of the total length of PIs (33397 bp) may be covered by H-NS. 3D models of H-NS binding sites, taken from independent genomic loci, were therefore analyzed. Black curves in panels D and F of Figure 5 show that their stacking energy and variability of **φ** are close to those of PIs, which is not typical for normal promoters. Deletion of *hns* increased transcription output from at least 4 investigated PIs (manuscript in preparation). Thus we concluded that H-NS is involved in interaction with PIs, and structural properties of its binding sites contribute to the specific characteristics of *promoter islands*.

**Table 1:** Association of *promoter islands* with horizontally acquired genes and H-NS.

Location of PIs		Association^1^					H^2^	Location of PIs		Association^1^					H^2^
5′-end_length	Genes	A	B	C	D	E		5′-end_length	Genes	A	B	C	D	E	
29150_313	*dapB*(+)/*carA(+)*						G	2882192_323	*casA(−)/cas3(−)*	+	+	1	1		K
83898_426	*leuL(−)/leuO(+)*		+	1			A	2901670_347	*ygcE(+)/queE(−)*		+			1	A
121694_343	*aroP(−)/pdhR(+)*					1	G	2903475_338	*queE(−)/ygcG(+)*		+	1	1	1	K
156927_379	*yadN(−)/folK(−)*		+	1	1		A	2986202_358	*yqeH(+)/yqeI(+)*		+	1	1		A
310529_398	*ecpR*(−)/*ykgL*(+)	+		2		1	A	2988974_339	*ygeF(+)/ygeG(+)*		+	1	1	1	A
383994_309	*yaiS(−)/tauA(+)*			1	1	1	A	2989603_468	*ygeG(+)/ygeH(+)*		+	1	1	1	A
522099_304	*ybbP(+)/rhsD(+)*		+	1	1	1	A	2991357_340	*ygeH(+)/ygeI(+)*		+	1	1		A
557105_351	*folD(−)/sfmA(+)*	+		1	1	1	K	2992989_612	*ygeK(−)/ygeM(−)*		+	2	2		A
576129_354	*nmpC(−)/essD(+)*	+	+		2		A	3117080_374	*yghJ(−)/glcA(−)*					1	A
582438_1016	*tfaX(+)/appY(+)*	+	+	1	1		A	3265097_477	*tdcA(−)/tdcR(+)*		+	2	2		K
583602_323	*appY(+)/ompT(−)*	+	+	2	2		A	3266734_706	*yhaC(+)*		+	1	1		A
584821_351	*ompT(−)/envY(−)*	+	+	1	1		A	3285165_325	*agaI(+)yraH(+)*		+	1	1	1	K
751980_413	*ybgD(−)/gltA(−)*		+	1	1		A	3383263_333	*argR(+)/yhcN(+)*				1		O
953696_434	*focA(−)/ycaO(−)*		+				K	3453428_392	*gspA(−)/gspC(+)*			2	1		K
996773_328	*ssuE(−)/elfA(+)*			1	1		A	3580023_317	*yhhZ(+)*		+	1	1		K
1196665_422	*ymfD(−)/ymfE(−)*	+	+	1	1	1	A	3581031_347	*yrhA(+)*		+	1	1		K
1210318_317	*mcrA(+)/icdC(+)*	+	+	1	1	1	A	3631905_345	*yhiL(−)*		+	1	1		A
1255333_301	*ycgV(−)ychF(−)*			1			A	3632424_449	*yhiL(−)/yhiM(+)*		+	2	2	1	K
1332795_340	*cysB(+)/ymiA(+)*						K	3648929_470	*arsC(+)/yhiS(+)*		+	1	1		A
1432784_339	*ynaE(−)/uspF(−)*		+	1	1		O	3651288_639	*insH_11(−)/slp(+)*		+	1	2	1	K
1463061_385	*paaY(+)/ydbA(+)*		+			1	K	3767592_418	*yibV(+)/yibH(−)*		+				A
1527917_612	*ydcD(+)/yncI(+)*		+	1	1		A	3794947_496	*waaC(+)/rfaL(+)*	+	+	1	1		A
1570060_392	*gadB(−)/pqqL(−)*						A	3797063_551	*waaK(−)/rfaZ(−)*	+	+	1	1	1	A
1581576_327	*ydeO(−)/safA(−)*	+	+	1	1		A	3798731_723	*waaY(−)/waaJ(−)*	+	+	1	1	1	A
1596197_345	*ydeK(−)/lsrK(−)*			1			A	3802145_1102	*waaB(−)/waaP(−)*	+	+	2	2	2	A
1636643_433	*cspI(−)ydfP(−)*	+	+		1		A	3834632_331	*selC(+)/setC(+)*			1			A
1752593_318	*ydhY(−)/ydhZ(−)*		+	1			A	3920739_440	*atpI(−)/rsmG(−)*			1			K
1811053_320	*ydjO(−)/cedA(−)*			1	1		K	4000528_663	*yigF(−)/yigG(−)*			1	1		A
1868534_304	*yeaI(+)*		+	1	1		A	4219964_389	*arpA(−)*		+	1	1		A
1903241_302	*yobD(+)/mntP(+)*			1		1	K	4248719_304	*malM(+)/yjbI(+)*		+	1	1	1	A
2054637_373	*amn(+)/yeeN(+)*	+	+	1	1	1	K	4249440_561	*yjbI(+)*		+	1	1	1	K
2101895_370	*wbbK(−)*	+	+	2	2	1	K	4258129_526	*zur(−)/yjbL(+)*		+	1	1		A
2190229_357	*yehD(−)/yehE(−)*	+	+	1	1		K	4266514_318	*tyrB(+)/yjbS(−)*		+			1	K
2342143_534	*yfaL(−)/ypaB(−)*		+	1		1	A	4280619_615	*yjcF(−)/actP(−)*		+	1	1	1	A
2363626_315	*ais(−)/arnB(+)*		+	1	1	1	K	4474585_660	*yjgL(+)*	+	+	1	1		A
2453647_489	*yfcV(−)/sixA(−)*		+				K	4537484_311	*nanC(−)/fimB(+)*	+	+	2	2	1	K
2461920_303	*yfdF(+)/mlaA(−)*			1	1		A	4539580_404	*fimB(+)/fimE(+)*	+	+	1	1		K
2467210_667	*yfdI(+)*	+	+	1	1	1	A	4540575_443	*fimE(+)/fimA(+)*	+	+		1	1	A
2468092_410	*yfdI(+)/tfaS(+)*	+	+	1	2	1	A	4554354_526	*yjiC(−)/iraD(+)*		+	2	1		K

1 “+” in columns **A** and **B** mark PIs overlapping with “*genomic islands*” found by IslandViever or GIST [33], respectively; in columns **C**–**E** - PIs associated with foreign genes predicted by Nakamura et al. [55] Lawrence et al [56] or Price et al. [57], respectively.

2 Letters in column “**H**” mark PIs interacting with H-NS. **G**: according to Grainger et al. [36], [37]; **K**: according to Kahramanoglou et al. (*E.coli* K12 MG1655) [35] and Oshima et al. in (*E.coli* K12 W3110) [52]; **O**: according to Oshima et al. [52] and Grainger et al. [36], [37]; **A**: according to all studies.

### *Promoter islands* are associated with horizontally acquired genes

The histone-like protein H-NS acts as a global repressor of transcription and preferentially suppresses the expression of horizontally-acquired genes [35], [52]–[54]. Thus we checked whether PIs are also associated with foreign genes using coordinates of long “*Genomic islands*” identified by IslandViewer [39] or GIST [33] as well as predictions made by Nakamura et al. [55], Lawrence and Ochman [56] and Price et al. [57] for individual genes. We found that 75 out of 78 PIs are associated with presumably foreign genes (Table 1). Sets of alien genes, predicted by five different approaches, overlapped with 24–63 *promoter islands*. Except GIST, the largest overlap was observed with genes, whose foreign origin was predicted on the basis of comprehensive sequence comparison or codon usage profiles [55], [56]. The numbers of PIs, associated with genes of these two sets were 3–4-fold greater than expected by chance. Thus, we concluded that PIs are associated with horizontally acquired genes and compared their functional and structural properties with “normal” promoters of alien genes. Promoters for this comparison were searched within the “*Genomic islands*” found by IslandViewer because foreign origin of long DNA fragments is predicted with a higher reliability than of single genes and IslandViewer makes predictions without relying on transcription signals.

Though A/T content of the selected promoters was almost the same as in the set of *multiple* promoters (58.9 and 58.3%, respectively), the average number of TSPs was 1.3-fold higher but 5-fold lower than in PIs. Nineteen promoters of “*Genomic islands*” gave perfectly matching samples in the analyzed data set of sequence reads [22], which is 2.7-fold less than that given by *multiple* promoters, but ∼2-fold greater than contribution given by PIs. Finally, 65.4% of *alien* promoters interacted with H-NS, which is also an intermediary between *multiple* promoters (46.9%) and PIs (100%). Thus, promoters of alien genes are more similar to PIs than promoters of two other groups.

The similarity to PIs increased when we removed twenty promoters, which activity profiles were typical for normal promoters: produced long RNAs more efficiently than short oligos (indicated in Table S1). However, the transcriptional output of the remaining 58 promoters was low both in the range of long RNAs and short oligos (Figure S1). A heightened amount of short products (Figures 3, 4 and S1) may therefore be considered as a specific property of PIs. In terms of structural metrics the molecular models of the set of *alien* promoters were intermediate between *multiple* promoters and PIs (Figures 5 and S2). But in stacking energy they were the same as PIs and differ significantly from normal promoters (panels F in Figures 5 and S2).

## Discussion

We found that in the genome of *E.coli* there are at least 78 sites with an extremely high density of potential promoters, which can produce short oligos, but the synthesis of normal mRNAs is suppressed. A contribution of *promoter islands* to the population of short RNAs is particularly evident for 9–10 bases long oligonucleotides (Figures 3, 4 and S1). Most probably these short RNAs are just by-products of the arrested transcription complexes. On the other hand, it can not be excluded that they prime RNA synthesis from some promoters [18], form complementary duplexes with cellular RNAs targeting them for hydrolysis [48], or interact with DNA template keeping *promoter islands* in a quasi-open state. In any case it is clear that the genomic DNA of *E.coli* contains regions with unusual transcriptional output.

Analysis of the available chip-on-chip data (Figure 6, Table 1) indicated that PIs provide a platform for interaction with both RNAP and H-NS. Oshima et al. [52] presumed that the formation of ternary complexes RNAP-H-NS-DNA is a general mode of transcription repression by H-NS. Simultaneous binding of RNAP and H-NS may therefore be crucial for transcription silencing. It could not be excluded, however, that normal transcription of PIs can be restored in some conditions. Thus, the presence of salicylic acid, which down regulated most genes [36], increased the percentage of normal promoters interacting with H-NS but 2-fold reduced it for PIs (data not shown). Structural or functional remodeling of PIs may therefore be required for the bacterial survival under stress condition. On the other hand, the contribution of PIs may be limited to a simple release of protective proteins, which seem to accumulate within the *islands*.

We found that 75 out of 78 *islands* are associated with presumably foreign genes (Table 1). Three other PIs are located upstream of genes encoding the small subunit of amidotransferase (CarA), the β-subunit of glutamate decarboxylase (GadB) and uncharacterized protein (YmiA). All these genes are transcribed normally, but the expression of *carA* and *gadB* is subjected to a very complex regulation involving 5–6 transcription factors, that can probably compensate for the negative impact of unusual genetic environment. It was proposed previously [33] that excessive promoters in “*Genomic islands*” were emerged by accelerated evolution of regulatory regions so as to integrate foreign genes into the host cells regulatory networks. Our present data are consistent with this hypothesis. Although we found that the number of potential TSPs as well as most structural metrics change in the order *single promoters*→*multiple promoters*→*promoters of alien genes*→PIs, while the transcriptional activity decreases in order *multiple promoters*→*single promoters*→*promoters of alien genes*→PIs. Thus, excessive promoters does not necessarily guarantee an active transcription and bacterial population may use them just in opposite manner so as to maintain regulatory regions of horizontally acquired genes in the heterochromatin-like state.

**Figure 7:**
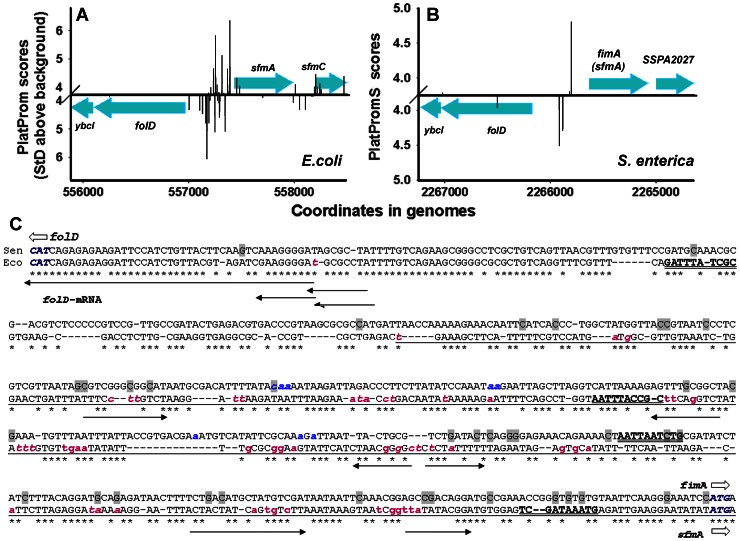
Distribution of potential promoters within regulatory region of *folD*-*sfmA*(*fimA*). Potential TSPs predicted in the genomes of *E.coli* K12 MG1655 (Eco) and *Salmonella enterica serovar Paratyphi* A str. AKU_12601 (Sen) by PlatProm [27] and PlatPromS [33] are shown on panels **A** and **B**, respectively. The panel **C** shows alignment of nucleotide sequences of corresponding intergenic spaces. Initiating codons of *folD* and *sfmA*(*fimA*) are indicated by open arrows and colored. Nucleotide sequence of PI in the genome of *E.coli* is underlined. PlatProm and PlotPromS predicted TSPs are shown by lower-case letters (magenta and blue, respectively). For the *folD* direction they were printed in italics. H-NS binding modules found by the *Virtual Footprint* [38] software (scores 5.43–6.29) are indicated in bold and double underlined. Registered sequence reads [22] are indicated by black arrows. Putative sites of deamination are shaded.

In any case we assume that excessive promoters evolve in the recipient genome after the transfer. If so, the regulatory regions of transferred genes in genomes of their donors should have normal promoters. Using the BLAST NCBI, we found potential donors for several genes associated with PIs and observed that this is really the case. Thus, for instance, certain homology (query cover 83–86%, identity 66–68%, E-value: 2e-35 - 2e-25) with alien gene *sfmA* [39], [55]–[57] encoding precursor for the type 1 fimbrial protein, was found only in the genomes of *Escherichiae*, closely related *Shigellas*, within practically all sequenced genomes of *Salmonella enterica* (gene *fimA*) and within three genomes of *Enterobacter cloacae*. *S. enterica* thus may be considered as a putative donor. We used PlatPromS version of our software previously adapted to the context of *Salmonella* promoters [33] to scan *folD* - *fimA* region in many strains of *S. enterica* and found that all of them contain normal promoters (exemplified in Figure 7B), while all tested genomes of *E.coli* have promoter dense region between *folD* and *sfmA* (Figure 7A). Therefore we propose to consider *promoter islands* as products of adaptive evolution.

The ability to receive and assimilate foreign genes is a feature of bacterial evolution (review: [58]). Molecular mechanisms of the transfer (transformation, transduction and conjugation) are well known. There are several approaches able to find foreign DNA [33], [39], [55]–[57]. But the mechanisms adapting foreign genetic material to the regulatory networks of novel host are not clear. The observed association of horizontally acquired genes with promoter-dense regions provides an opportunity for targeted research. Even though the force driving accumulation of promoter-like signals or H-NS binding sites near the foreign genes remains obscure, comparative analysis of *promoter islands* with regulatory regions of potential donors revealed certain symptomatic features, which are exemplified in Figure 7C. Thus, alignment of *folD*-*sfmA*(*fimA*) intergenic sequences from the genomes of *S.enterica* and *E.coli* by T-Coffee [59] besides insertions and deletions, which compensated a 64 bp difference in length, suggested 161 point mutations; 135 of them lie within the *promoter island* (underlined). Thirty-four substitutions decreased the AT content in this region, 47 were neutral, and 54 mutations increased it. Thus the total AT-content in the region flanking *sfmA* gene became higher than in the promoter area of *fimA* (61.8% and 68.5, respectively), which is typical for alien DNA and PIs. Thirty-five of 54 substitutions (64.8%) that increased AT content can appear as a result of cytidine deamination (shaded nucleotides in Figure 7C). This reaction is catalyzed by the enzyme cytidine/deoxycytidine deaminase, converting cytidine (deoxycytidine) and metilcytidine in uridine, deoxyuridine and thymine, respectively. The primary function of *E.coli* cytidine deaminase (gene *cdd*) is to produce uridine. So far it is not clear, whether this enzyme can modify cytidines within the DNA or RNA molecules, though in 3D structure it is related to the human cytidine deaminase specifically editing ApoB RNA (APOBEC-1) [60] and modifying cytidines within DNA [61]. On the other hand, mammalian APOBEC-1 is related to the key enzyme diversifying antigen receptor gene in B lymphocytes (Activation-induced cytidine deaminase, AID) [62] and playing an important role in immune response. It has been recently demonstrated that AID targets DNA at single stranded sites of RNAP II stalling complexes [63], [64]. If Cdd or another bacterial enzyme can deaminate cytidines within single stranded DNA, than we suggest the following model for rapid evolution of PIs.

Evolutionary conservatism of bacterial transcriptional machinery in most cases allows the host RNAP to recognize promoters of horizontally transferred genes. But the lack of suitable activators or collision with foreign genetic environment may detain the transcription complex on the promoter exposing cytidines within the transcriptional bubble for deamination. As a result, promoter regions of foreign genes will gradually accumulate the T/A and A/T base pairs, creating H-NS binding sites (consensus in *E.coli* TCGATAAATT [65]) with a high probability. In the case of PI from the regulatory region of *sfmA* at least two H-NS binding sites were evolved (Figure 7C). The ternary complex DNA-RNAP-H-NS formation [52] or oligomerization of H-NS on A/T-reach DNA [66] should stabilize arrested state of the complex, leading to further accumulation of A/T-pairs and formation of the *promoter island*. Synthesis of at least 2 of 6 short RNAs derived from this PI can be blocked by H-NS (Figure 7C). Since halted transcription complexes can sometimes be formed within coding sequences, some *promoter islands* lie within genes (Figure 1) thus contributing to the well known abundance of foreign genetic material with A/T base pairs. Repressing transcription of useless or toxic genes *promoter islands* also create the conditions for the possibility of their expression in the changed environment and offer a set of suitable promoters to integrate beneficial genes into regulatory networks of novel host. *Promoter islands* thus can be considered as special instruments of evolution used by bacterial population in order to acquire the foreign genetic material.

## Supporting Information

Figure S1 **Relative amount of RNAs of different lengths in the cells of** ***E.coli*** **K12 MG1655.** Samples containing at the 5′-end sequences matching the genomic DNA for the indicated length were collected step by step, as described in Methods and in the text. At the first 17 steps matching samples were collected from the whole set of registered sequence reads [22] (shaded area), while at the steps 18–36 – from samples with adapter sequence at the 3′-end. For *alien* promoters samples were collected within ±50 bp regions surrounding TSPs; for each PI – within the area covered by the *island* and ±50 bp flanking regions. In the case of *multiple* promoters analyzed areas included the genomic regions located between the first and the last TSPs, as well as 50 bp flanking sequences. The number of samples, collected at each step for a particular set of genomic regions, was normalized per the total number of sequence reads analyzed at this step, and per the total length of genomic regions in a set. In the case of random distribution it will give a value equal to 1.0 (dashed line). To increase the resolution in the bottom part of the figure we changed the scale of the Y-axis at the level 0.54.
(TIF)

Figure S2 **Histograms representing results of structural analysis for** ***multiple*** **promoters and promoters of** ***alien*** **genes.** Metric parameters (specified under the X-axes) were obtained using the *carbon* (**B**, **C** and **D**) or *phosphorus* (**A**, **E**, and **F**) *chains*. Studied genomic regions and the colors used are indicated in the panel **A**. The number of fragments that have similar values of the measured parameters were combined in the intervals “***i***”, which are indicated in panels. Parameters **RL-SL**, **Ω** and stacking energy were measured for fragments of different lengths in the range 20–200 bp. Observed dependences were exemplified for fragments of indicated length (**S**). The numeric values obtained for 58 promoters of horizontally acquired genes were normalized to the size of other sets. Molecular models of *single* and *alien* promoters were created for sequences lying around the transcription start points (between positions −150 and +149).
(TIF)

Table S1 Genomic coordinates of *promoter islands* and other selected genomic regions.
(XLS)
