# Supplementary figures and images for: Promoters of Escherichia coli versus Promoter Islands: Function and Structure Comparison
Source: PLoS One. 2013 May 22;8(5):e62601. doi: 10.1371/journal.pone.0062601 (PMC3661553; doi:10.1371/journal.pone.0062601)

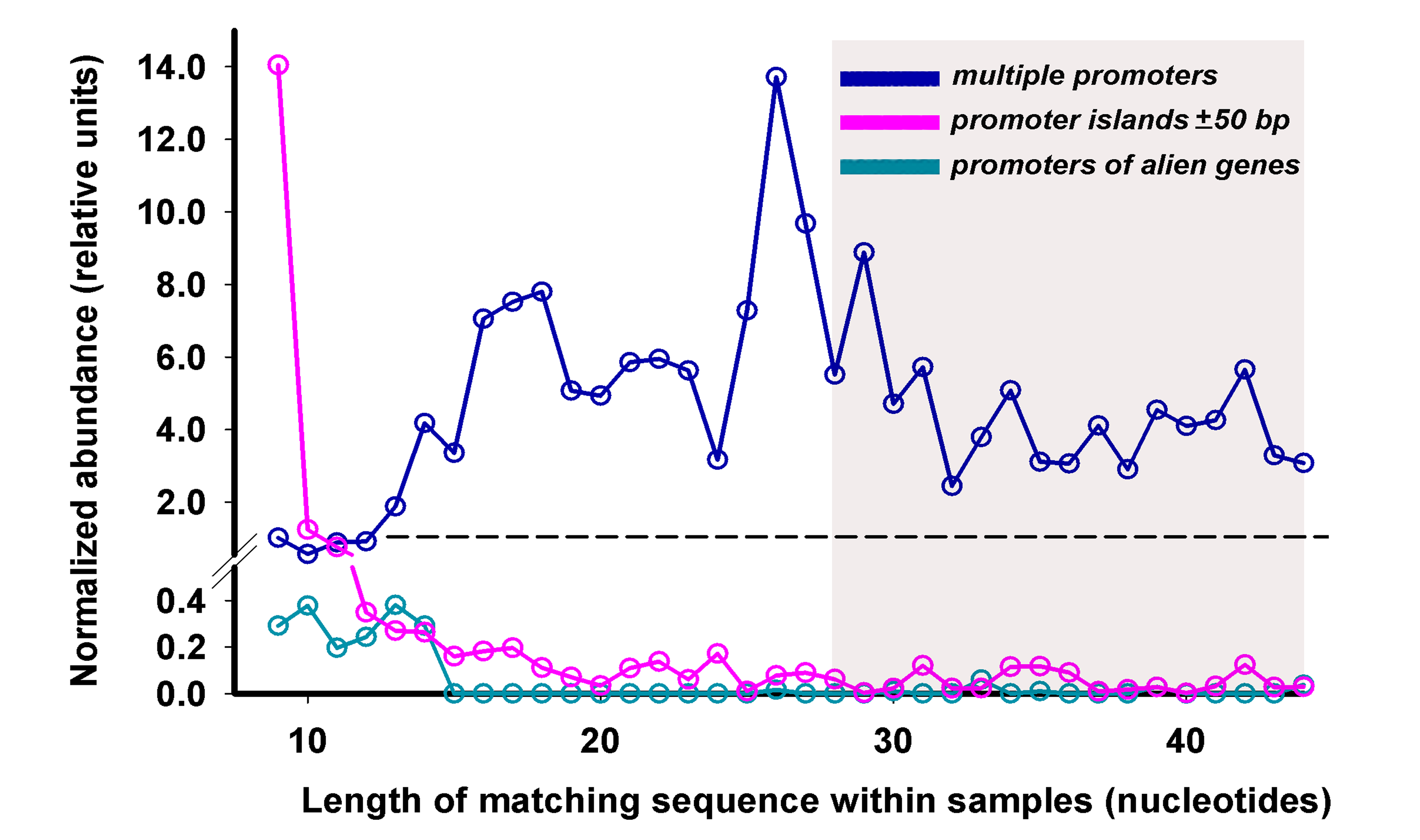

Supplement: Figure S1 — Relative amount of RNAs of different lengths in the cells of E.coli K12 MG1655. Samples containing at the 5′-end sequences matching the genomic DNA for the indicated length were collected step by step, as described in Methods and in the text. At the first 17 steps matching samples were collected from the whole set of registered sequence reads [22] (shaded area), while at the steps 18–36 – from samples with adapter sequence at the 3′-end. For alien promoters samples were collected within ±50 bp regions surrounding TSPs; for each PI – within the area covered by the island and ±50 bp flanking regions. In the case of multiple promoters analyzed areas included the genomic regions located between the first and the last TSPs, as well as 50 bp flanking sequences. The number of samples, collected at each step for a particular set of genomic regions, was normalized per the total number of sequence reads analyzed at this step, and per the total length of genomic regions in a set. In the case of random distribution it will give a value equal to 1.0 (dashed line). To increase the resolution in the bottom part of the figure we changed the scale of the Y-axis at the level 0.54. (TIF) [file pone.0062601.s001.tif]

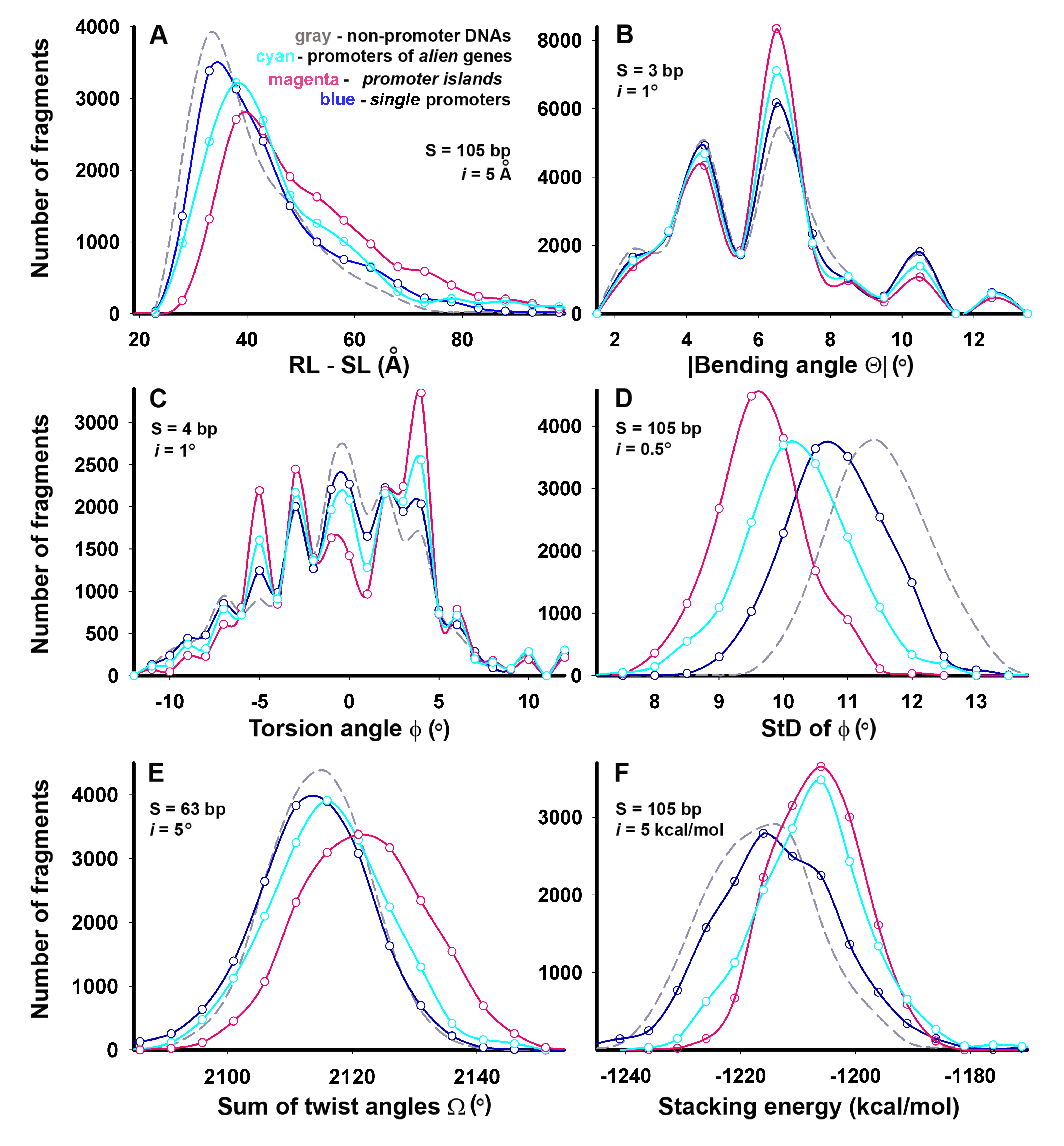

Supplement: Figure S2 — Histograms representing results of structural analysis for multiple promoters and promoters of alien genes. Metric parameters (specified under the X-axes) were obtained using the carbon (B, C and D) or phosphorus (A, E, and F) chains. Studied genomic regions and the colors used are indicated in the panel A. The number of fragments that have similar values of the measured parameters were combined in the intervals “i”, which are indicated in panels. Parameters RL-SL, Ω and stacking energy were measured for fragments of different lengths in the range 20–200 bp. Observed dependences were exemplified for fragments of indicated length (S). The numeric values obtained for 58 promoters of horizontally acquired genes were normalized to the size of other sets. Molecular models of single and alien promoters were created for sequences lying around the transcription start points (between positions −150 and +149). (TIF) [file pone.0062601.s002.tif]
